# Ovarian Cyst Enlargement in a 14 Year Old Female with Persistent Ascities, Severe Hypothyroidism and Elevated Serum CA-125 Level

**Published:** 2012-06-30

**Authors:** F Motamed, K Eftekhari, M A Kiani, A Rabbani

**Affiliations:** 1 Department of Gastroenterology, Children's Medical Center Hospital, Tehran University of Medical Sciences, Tehran, Iran; 2 Department of Pediatrics, Mousavi Hospital, Zanjan University of Medical Sciences, Zanjan, Iran; 3 Department of Endocrinology, Children's Medical Center Hospital, Tehran University of Medical Sciences, Tehran, Iran

**Keywords:** Ovarian cyst, Hypothyroidism, CA-125, Van Wyk and Grumbach syndrome

## Abstract

A 14 year old female complained of abdominal pain and distention with vomiting. The physical exam showed thyroid enlargement and ascites. The imaging evaluation demonstrated a large ovarian cyst. Laboratory tests depicted hypothyroidism and marked elevation of Carbohydrate antigen 125 (CA-125) levels. As the bone age was 10 years, more retarded than the chronological age, Van Wyk and Grumbach syndrome was suspected. Treatment with thyroid hormone was initiated and the condition improved dramatically with disappearance of symptoms and signs 5 weeks later.

## Introduction

Hypothyroidism is a common endocrine disorder. Some patients with severe hypothyroidism show rare clinical features such as coma, pericardial, pleural and peritoneal effusions. However, ascites is an uncommon feature occurring in only 4% of patients.[1] If hypothyroidism is not appropriately treated, after few years, it could cause ovarian cystic enlargement. This association was first reported in 1960 by Van Wyk and Grumbach, so named Van Wyk and Grumbach syndrome.[2] Although this syndrome is very rare, and surgery for ovarian cysts is not necessary, sometimes inadvertent surgery has been performed due to the lack of information about this disease.[3] Carbohydrate antigen 125 (CA-125) is one of the tumor markers related to ovarian malignancy and shows the presence of tissue related to the coleomic epithelium.[4][5] Benign ovarian neoplasms, several disorders of uterus , liver, gallbladder, pancreas, gastrointestinal tract and hypothyroidism are non-malignant causes of elevated serum CA-125 levels.[6]

## Case Report

A 14 year old female complained of diffuse abdominal pain and distention with vomiting. These problems started suddenly three days before referring to our clinic, without any history of disease. The abdominal pain was not associated with meals. Nausea and vomiting were present too. She was referred to our clinic for further evaluation and was admitted at the Gastroenterology Ward. Past medical history was unremarkable. The family history showed that the parents were non-consanguineous and the mother had epilepsy. At physical examination, she had a puffy face and enlargement of the thyroid gland palpable in the neck. Vital signs were normal. Lungs were clear and heart sounds were normal too. The abdomen was distended with shifting dullness (due to ascites) without organomegalies. Extremities were normal without edema or deformities. Her pubertal development was appropriated for age; however (Stage II Public and III Tanner) bone age was 10 years, more retarded than the chronological age.

Laboratory routine data including FSH and LH were within normal limits. Tumor markers tests showed strong elevation of serum CA-125 levels (105.4 u/ml) but CEA was normal. Thyroid function tests revealed very marked elevation of TSH -more than 100 mu/L, T4 and T3 depicted very low levels, 0.5 mu/L (with lower limit normal 8) for T3 and 25 mu/L (with lower limit normal 100) for T4 respectively.

Abdomino-pelvic ultrasound and CT scan showed a cystic mass with a diameter of 40×45 mm located at the left lower quadrant (LLQ) which originated from the left ovary. The uterus was pubertal size (55×21 mm) with normal endometrial echo. Moderate free fluid was present in the peritoneal cavity. Liver, gallbladder, spleen, pancreas and kidneys were normal in structure and echo.

Because of elevation of serum CA-125 levels, ovarian malignancy was included in differential diag-noses, but as Van Wyk and Grumbach syndrome was the main diagnosis, thyroxin (levothyroxin) replacement was started at a dose of 50 µg per day and gradually increased weekly up to 150 µg per day. After 4 weeks of treatment, the ovarian cyst was smaller and the ascites disappeared. In the laboratory examination not only TSH, T_4_ and T_3_ reached the normal range, but also CA-125 normalized too. The follow-up ultrasound after 5 months of initiation of therapy revealed complete regression of the ovarian cyst and the patient was well without any complications.

## Discussion

Van Wyk and Grumbach described a syndrome for first time consistent of hypothyroidism, precocious puberty and multicystic ovaric enlargement with regression of the cyst and reversal to prepubertal stage after thyroid hormone replacement.

In our case, differentiation between ovarian cancers from benign etiology was important. The clinical features and laboratory findings suggested severe hypothyroidism. Elevation of CA-125 levels is caused by this disease too. Ascites is a rare complication of hypothyroidism (4%). Association of hypothyroidism, ascites (myxedema) and high levels of CA-125 is very rare as well.[7] Elevation of CA-125 probably is due to delay of clearance of CA-125 secondary to hypothyroidism,[8] presence and severity of pericardial effusion,[9] peritoneal inflammation caused by ascites[10] or increased secretion of this marker by the ovarian cysts.[11]

After initiation of thyroid hormone treatment, her symptoms disappeared, ascites completely reabsorbed and CA-125 normalized.

In summary, hypothyroidism should be included in the work-up of patients with ovarian cysts, especially if associated with high level CA-125 markers, in order to avoid unnecessary surgery.
